# Supplementary material for: Viral vector-mediated reprogramming of the fibroblastic tumor stroma sustains curative melanoma treatment
Source: Nat Commun. 2021 Aug 5;12:4734. doi: 10.1038/s41467-021-25057-w (PMC8342618; doi:10.1038/s41467-021-25057-w)
Supplement: Supplementary file 1 — Supplementary Information File [file 41467_2021_25057_MOESM1_ESM.pdf]

Supplementary Information

Viral vector-mediated reprogramming of the fibroblastic tumor stroma sustains curative melanoma treatment

Ring et al.

Ring et al., Supplementary Figure 1

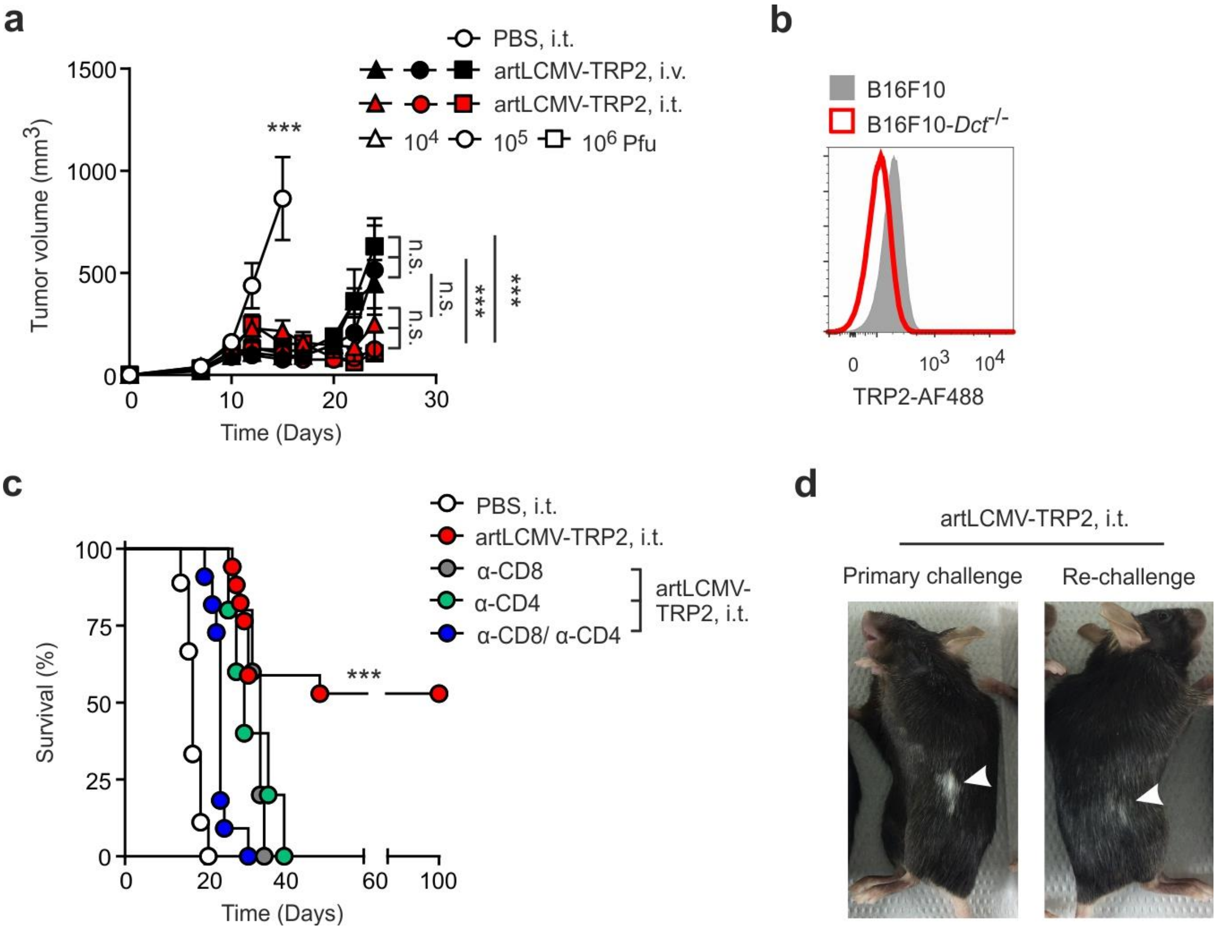

Supplementary Figure 1. Tumor rejection after i.t. artLCMV-TRP2 treatment depends on TRP2-specific T cells. a, Tumor growth kinetics of mice treated i.v. or i.t. with different doses of artLCMV-TRP2 . Dots indicate mean±s.e.m. for each time point. **b**, Flow cytometric analysis of TRP2-deficient B16F10 cells (B16F10-*Dct*$^{-/-}$). **c,** Survival of mice treated i.t. with artLCMV-TRP2 and injected with CD8- or CD4-depleting antibodies. **d**, Mice developed vitiligo-like fur depigmentation after i.t. treatment with artLCMV-TRP2 at the side of tumor inoculation. Data from one experiment with n=4 (PBS; 10^4 artLCMV-TRP2, i.v., 10^5 artLCMV-TRP2, i.v., 10^5 artLCMV-TRP2, i.t., 10^6 artLCMV-TRP2, i.v.), n=5 (10^4 artLCMV-TRP2, i.t.) and n=6 (10^6 artLCMV-TRP2, i.v.) mice (a). Data from one experiment with n=5 (artLCMV-TRP2, i.t.+α-CD4; artLCMV-TRP2, i.t.+α-CD8) and pooled data from two independent experiments with n=9 (PBS), n=17 (artLCMV-TRP2, i.t.) and n=11 (artLCMV-TRP2, i.t.+α-CD8/ α-CD4) mice (c). Statistical analysis was performed using two-way analysis of variance (ANOVA) with Bonferroni (a), Log-Rank Mantel-Cox test (c). *P < 0.05: **P < 0.01; ***P < 0.001. Source data and exact P values are provided in the Source data file.

Ring et al., Supplementary Figure 2

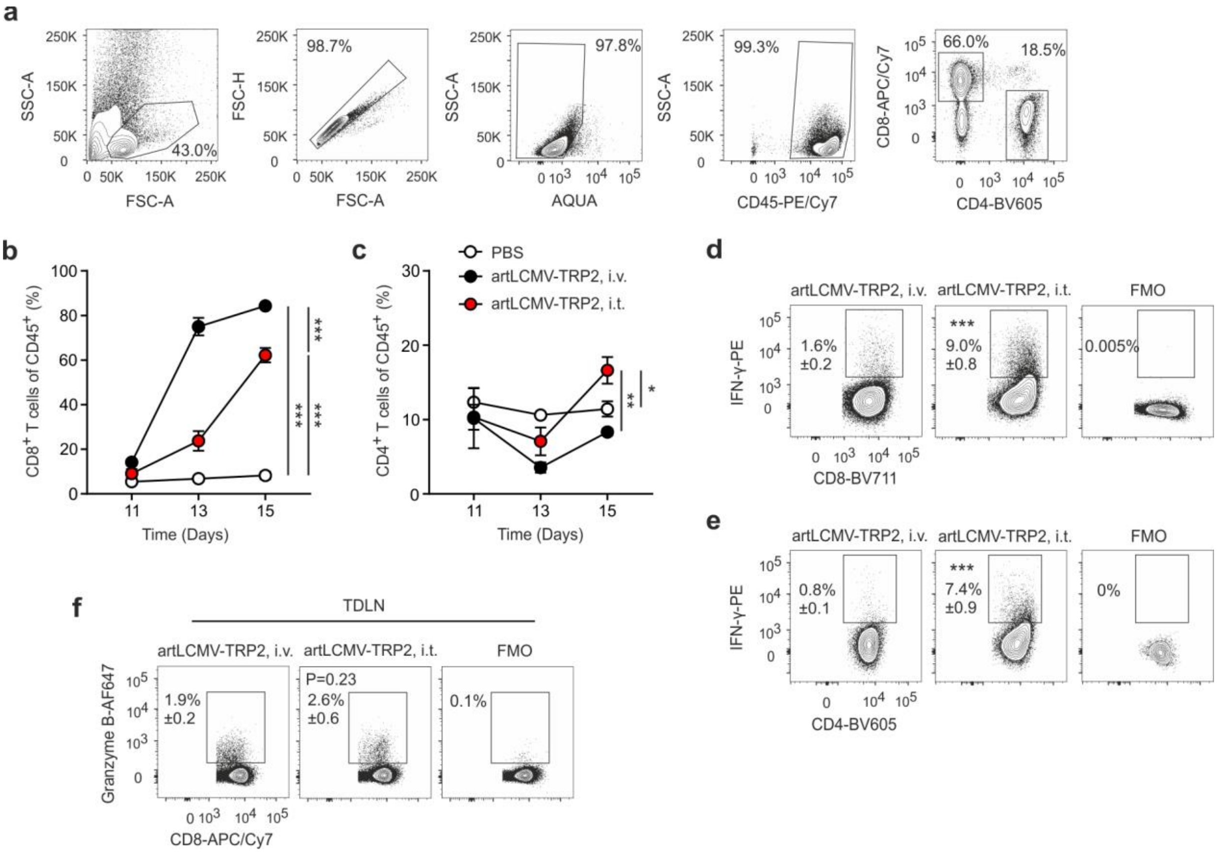

Supplementary Figure 2. Intratumoral artLCMV-TRP2 sustains the functionality of tumor-infiltrating T cells. B16F10-tumor bearing mice were immunized on day 7 with artLCMV-TRP2 i.v. or i.t. and tumor-infiltrating T cells were analyzed. **a,** Flow cytometry-based gating strategy for tumor-infiltrating T cell analysis used in Fig. 2a and b. **b and c**, Frequency of $CD8^+$ (b) and $CD4^+$ (c) T cells at indicated time points. Dots indicate mean±s.e.m. for each time point. **d and e,** Flow cytometry-based gating strategy for for IFN-γ expression in tumor-infiltrating $CD8^+$ (d) and $CD4^+$ (e) T cells on day 15 used in Fig. 2c. **f,** Representative plots for Granzyme B expression in $CD8^+$ T cells in the TDLN. Dots represent individual mice and lines indicate mean values. Pooled data from two independent experiments with n=4 (Day 11, PBS; Day 11, 13 and 15, artLCMV-TRP2, i.v.), n=6 (Day 11, artLCMV-TRP2, i.t.), n=7 (Day 13, PBS), n=8 (Day 15, PBS) and n=10 (Day 13, 15, artLCMV-TRP2, i.t.) mice (b, c), n=9 mice (d, f) and from one experiment with n=4 (artLCMV-TRP2, i.t.) and n=5 (artLCMV-TRP2, i.v.) mice (f). Statistical analysis was performed using one-way ANOVA with Tukey's multiple comparison test (b, c), or unpaired two-tailed Student`s t test (d-f) with $*P < 0.05$: $**P < 0.01$; $***P < 0.001$. Source data and exact P values are provided in the Source data file.

Ring et al., Supplementary Figure 3

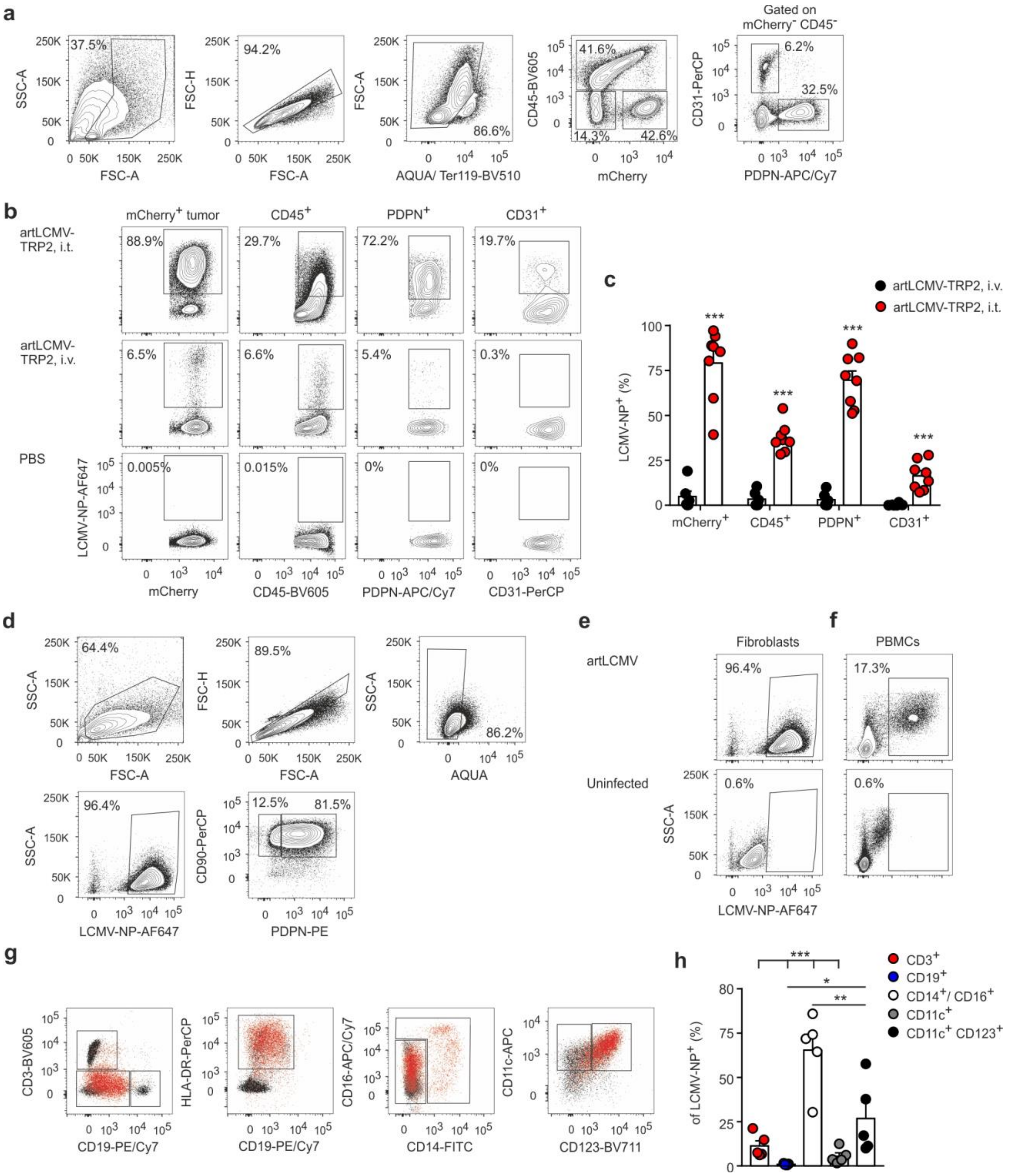

Supplementary Figure 3. artLCMV vectors transduce human and murine FSCs. a, Mice were injected with B16F10-mCherry and treated on day 7 i.t. or i.v. with artLCMV-TRP2. On day 11 tumors were harvested. Gating scheme for $mCherry^+$ tumor cells, $CD45^+$ immune cells, $PDPN^+$ FSCs and $CD31^+$ BECs isolated from the tumor used in Fig. 3c. **b-c,** Representative FACS plots (b) and frequencies (**c**) of LCMV-NP^+ cells in each cell subset in mice treated i.t. or i.v. with artLCMV-TRP2. **d-f,** Gating scheme for fibroblasts isolated from human skin and melanoma (d). Representative FACS plot for LCMV-NP staining after infection with artLCMV in (f) skin-derived fibroblasts and PBMCs used in Fig. 3f and g. **g,** Representative FACS Plot for PBMCs infected with artLCMV. Red dots indicate LCMV-NP^+ cells within each plot. **h,** Frequencies of $CD14^+$/ $CD16^+$ monocytes, $CD11c^+$ cDCs and $CD11c^+$ $CD123^+$ pDCs within LCMV-NP^+ cells. Dots indicate mean±s.e.m. Data from one experiment with n=6 (artLCMV-TRP2, i.v.) and pooled data from two independent experiments with n=8 (artLCMV-TRP2, i.t.) mice (c). Pooled data from n=5 patients (h). Statistical analysis was performed using unpaired two-tailed Student`s t test (c) or one-way ANOVA with Tukey's multiple comparison test (h) with *P < 0.05: **P < 0.01; ***P < 0.001. Source data and exact P values are provided in the Source data file.

Ring et al., Supplementary Figure 4

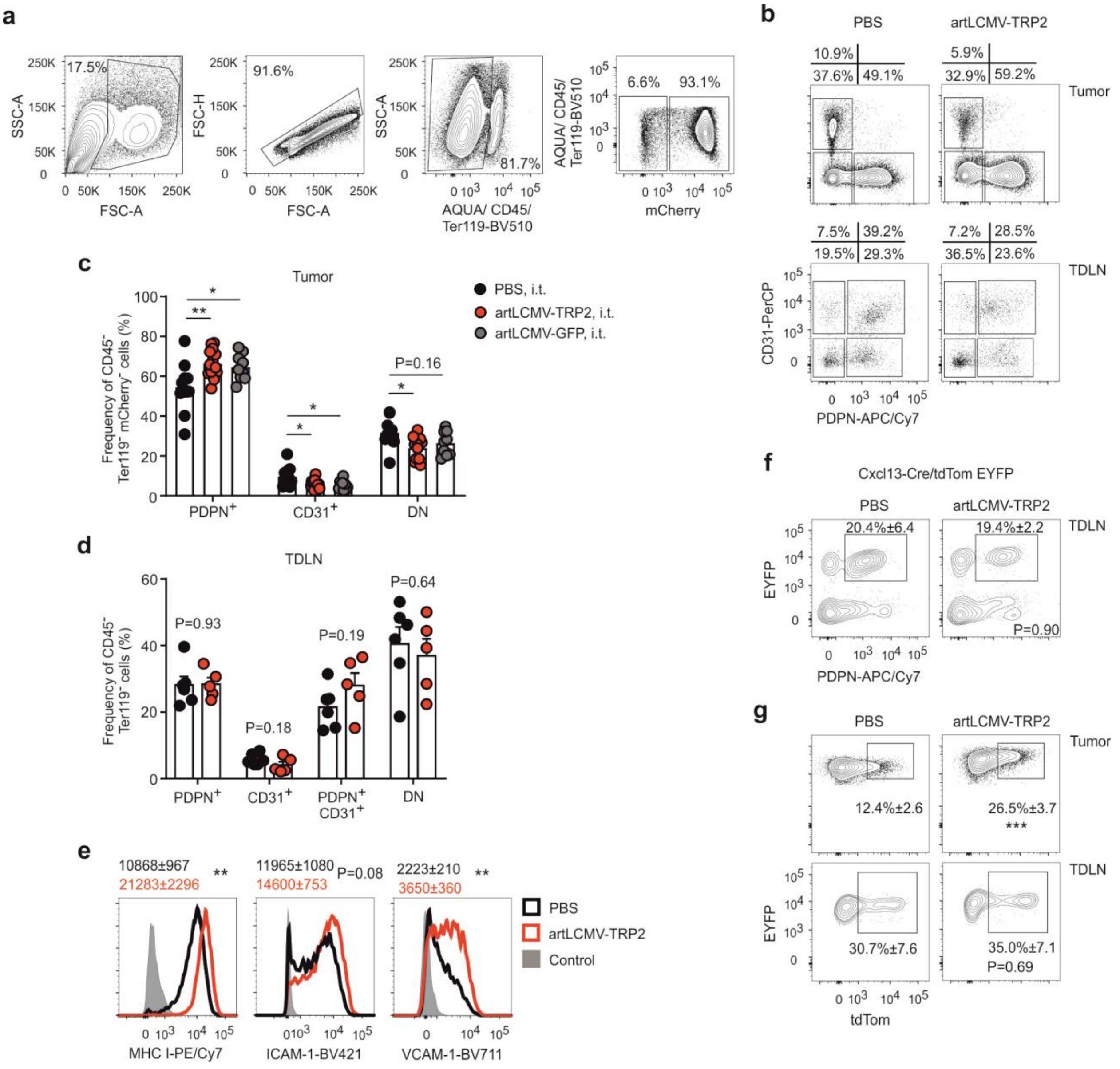

Supplementary Figure 4. Intratumoral artLCMV injection activates PDPN⁺ FSCs in the TME. B16F10-mCherry-tumor bearing mice were immunized with artLCMV-TRP2 or -GFP i.t. on day 7 and FSCs in tumor and TDLN were analyzed on day 11. **a,** Flow cytometry-based gating strategy for stromal cell analysis used in Fig. 4a. **b-d,** Representative FACS plots (b) and frequencies of stromal cell subsets in the tumor (c) and the TDLNs (d). Dots show mean±s.e.m. **e,** PDPN^{+} FSCs from the tumor were analyzed for the mean expression of the indicated marker. **f and g,** B16F10-mCherry cells were injected into Cxcl13-Cre/tdTom EYFP mice which were immunized i.t. on day 7 with artLCMV-TRP2. **f,** Representative FACS plots for EYFP^{+} PDPN^{+} FSCs in the TDLN. **g,** Analysis of current CXCL13 expression in EYFP^{+} PDPN^{+} FSCs as determined by the tdTomato reporter in the tumor as shown in Fig. 4f and in the TDLN. Values indicate mean±s.e.m. Pooled data from two independent experiments with n=11 (PBS, i.t.), n=14 (artLCMV-TRP2, i.t.) and n=9 (artLCMV-GFP, i.t.) mice (c); n=6 (PBS, i.t.) and n=5 (artLCMV-TRP2, i.t.) mice (d); n=7 (PBS) and n=6 (artLCMV-TRP2) mice (e); n=4 to 7 (PBS) and n=5 to 7 (artLCMV-TRP2, i.t.) mice (f, g). Statistical analysis was performed using unpaired two-tailed Student`s t test (c, d, and e-g) with *$P < 0.05$: **$P < 0.01$; ***$P < 0.001$. Source data and exact P values are provided in the Source data file.

Ring et al., Supplementary Figure 5

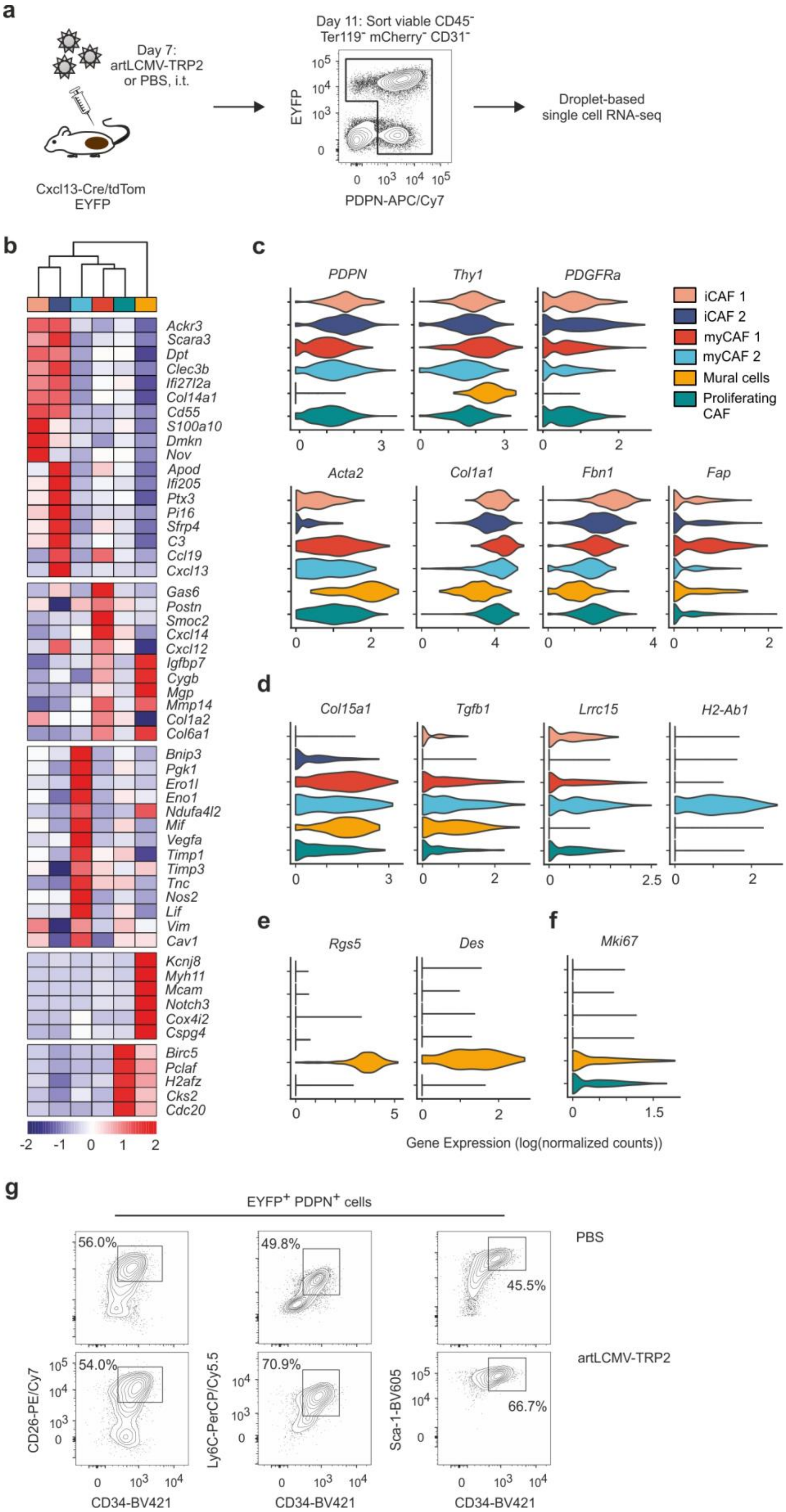

Supplementary Figure 5. Molecular landscape of tumor-associated EYFP^{+} FSCs. a, EYFP^{+} or PDPN^{+} FSCs were sorted from B16F10-mCherry tumors on day 11 following i.t. artLCMV-TRP2 or PBS application. **b,** Hierarchical clustering of averaged expression of cluster specific marker genes for tumor FSC clusters in merged PBS and artLCMV-TRP2. **c-f,** Violin plots showing gene expression for Pan-CAF marker genes (c), myCAF marker genes (d), mural cells (e) and proliferating FSCs (f). **g,** Representative flow cytometric plots of iCAF-specific marker CD26^{+} CD34^{+}, Ly6C^{+} CD34^{+} and Sca-1^{+} CD34^{+} cell populations within the EYFP^{+} PDPN^{+} cells used in Fig. 5d. Source data and exact P values are provided in the Source data file.

Ring et al., Supplementary Figure 6

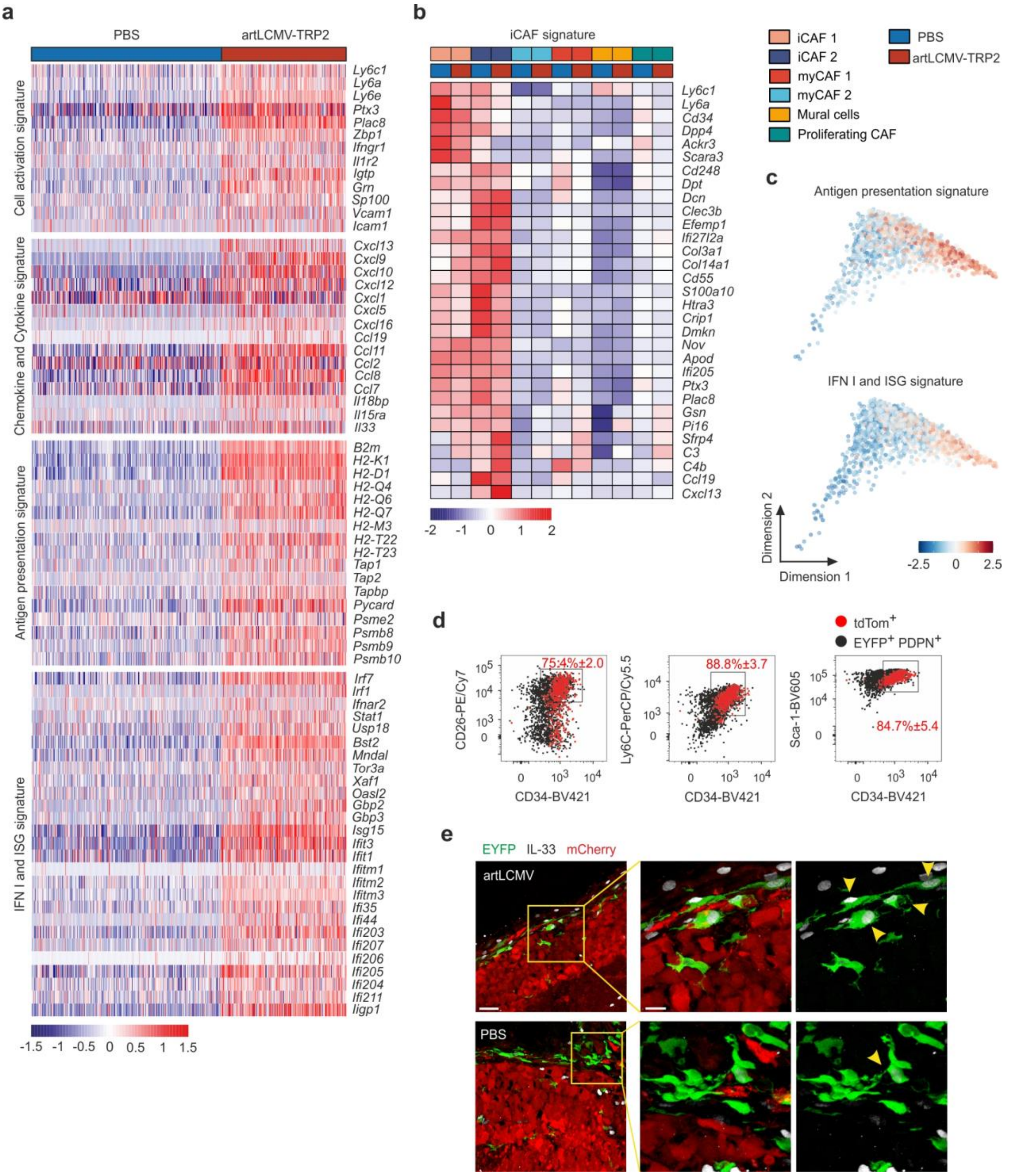

Supplementary Figure 6. Intratumoral artLCMV-TRP2 activates *Cxcl13*-expressing iCAF2 cluster with an inflammatory gene signature. a, DE gene analysis of iCAF1 and iCAF2 following i.t. artLCMV-TRP2 vs PBS control treatment. Heatmaps for selected DE genes which were assigned to distinct immune-stimulating pathways. **b,** Heatmaps displaying iCAF signature genes across all six identified CAF clusters in EYFP$^+$ FSCs merged from artLCMV and PBS-treated mice. **c,** Diffusion maps of EYFP$^+$ tumor FSCs with trajectories constructed based on differential genes analysis in clusters iCAF1 and iCAF2 between PBS and artLCMV condition. Diffusions maps showing antigen presentation and type I IFN and interferon-stimulating gene (IFN I and ISG) signatures. **d,** Flow cytometric analysis for tdTomato$^+$ cells showing current CXCL13 protein expression. Gates display CD26$^+$ CD34$^+$, Ly6C$^+$ CD34$^+$ and Sca-1$^+$ CD34$^+$ cell populations gated on EYFP$^+$ PDPN$^+$ cells. Values indicate mean±s.e.m. Pooled data from two independent experiments with n=6 mice (d). **e,** Representative confocal microscopy staining for IL-33 expression within EYFP$^+$ cells from n=3 (PBS) and n=4 (artLCMV) mice. Arrowheads indicate IL-33$^+$ EYFP$^+$ cells. Scale bar 40 μm (overview), 5 μm (boxed area). Source data and exact P values are provided in the Source data file.

Ring et al., Supplementary Figure 7

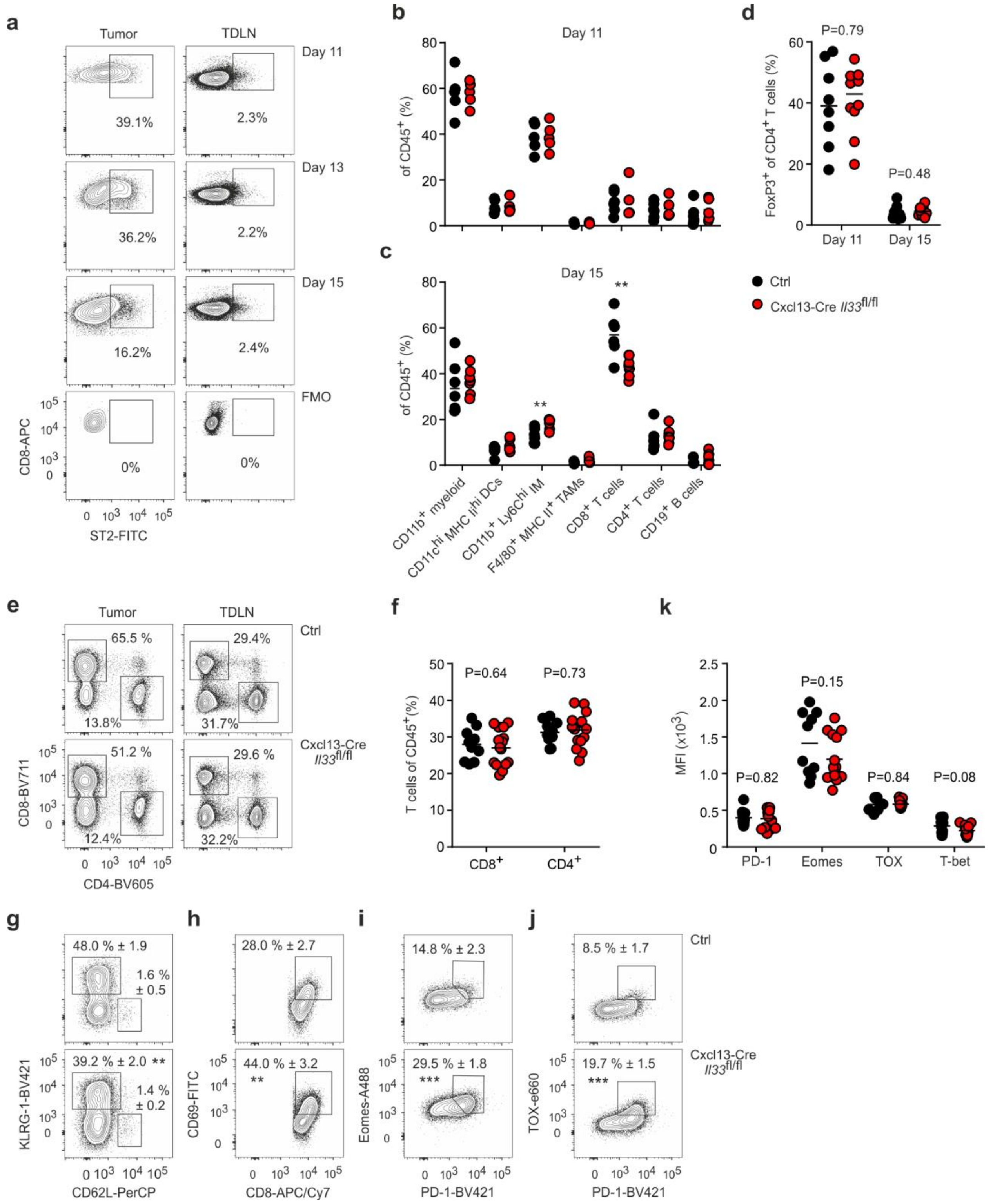

Supplementary Figure 7. Tumor FSC-derived IL-33 prevents exhaustion in tumor-infiltrating CD8$^+$ T cells. a, Mice were inoculated s.c. with B16F10 melanoma cells and treated on day 7 i.t. with artLCMV-TRP2. Representative FACS plots for ST2 staining on CD8$^+$ T cells in the tumor and TDLN at indicated time points as shown in Fig. 6a. **b-d**, Abundance of immune cell populations (b and c) and FoxP3$^+$ CD4$^+$ regulatory T cells (d) in the tumor on day 11 and day 15 in Cxcl13-Cre *Il33*$^{fl/fl}$ and Cre-negative littermate control (Ctrl) mice. **e and f,** Representative FACS plots of CD8$^+$ and CD4$^+$ T cells on day 15 in the tumor as quantified in Fig. 6d and the TDLN in Cxcl13-Cre *Il33*$^{fl/fl}$ and Ctrl mice. **f,** Pooled data showing frequency of CD8$^+$ and CD4$^+$ T cells in the TDLN. **g-j,** Representative FACS plots of KLRG1$^+$ CD62L$^-$ (g), CD69$^+$ (h), PD-1^{hi} Eomes^{hi} (i) and PD-1^{hi} TOX$^+$ (j) CD8$^+$ T cells in the tumor of Cxcl13-Cre *Il33*$^{fl/fl}$ and Ctrl mice as shown in Fig. 6d, h, i-k. **k,** Mean expression of Eomes, PD-1, TOX and T-bet on CD8$^+$ T cells in the TDLN. Dots represent individual mice and lines indicate mean values. Pooled data from two independent experiments with n=6 (Day 11, Ctrl) and n=5 (Day 11, Cxcl13-Cre *Il33*$^{fl/fl}$) mice (b); n=7 (Day 11, Ctrl and Cxcl13-Cre *Il33*$^{fl/fl}$) mice (c); n=7 (TOX, Ctrl) and n=11 (TOX, Cxcl13-Cre *Il33*$^{fl/fl}$) mice (k). Pooled data from three independent experiments with n=8 (Day 11, Ctrl), n=9 (Day 15, Ctrl) and n=10 (Day 11 and Day 15, Cxcl13-Cre *Il33*$^{fl/fl}$) mice (d); n=10 (Ctrl) and n=15 (Cxcl13-Cre *Il33*$^{fl/fl}$) mice (f); n=10 (PD-1, Eomes, T-bet, Ctrl) and n=15 mice (PD-1, Eomes, T-bet, Cxcl13-Cre *Il33*$^{fl/fl}$) mice (k). Statistical analysis was performed using unpaired two-tailed Student`s t test (b-d, f-k) with *P < 0.05: **P < 0.01; ***P < 0.001. Source data and exact P values are provided in the Source data file.

Ring et al., Supplementary Figure 8

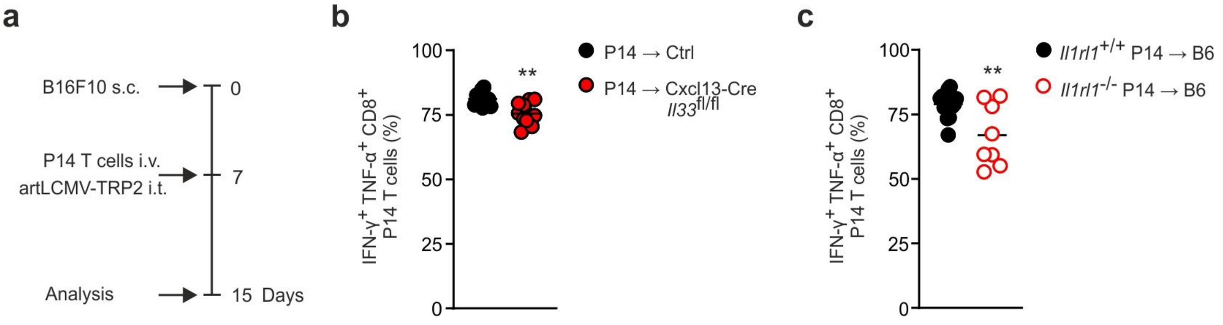

Supplementary Figure 8. Tumor FSC-derived IL-33 prevents exhaustion in tumor-infiltrating CD8$^+$ T cells. a, Mice were inoculated s.c. with B16F10 and CD45.1$^+$ P14 T cells (T cell receptor transgenic CD8$^+$ T cells specific for the LCMV epitope GP$_{33\text{-}41}$) were transferred i.v. into recipient mice on day 7. Recipient mice were subsequently treated i.t. with artLCMV-TRP2 and cytokine-expressing intratumoral CD8$^+$ P14 T cells were analyzed on day 15. **b**, Frequency of IFN-γ$^+$ TNF-α$^+$ CD8$^+$ P14 T cells transferred into Cxcl13-Cre *Il33*$^{fl/fl}$ and Cre-negative littermate control (Ctrl) mice. **c,** Frequency of cytokine$^+$ *Il1rl1* (ST2)-proficient or -deficient P14 T cells transferred into WT recipients. Dots represent individual mice and lines indicate mean values. Pooled data from three (b) or two (c) independent experiments with n=11 (Ctrl and Cxcl13-Cre *Il33*$^{fl/fl}$) mice (b); n=8 (*Il1rl1*$^{-/-}$ P14 → B6) and n=15 (*Il1rl1*$^{+/+}$ P14 → B6) mice (c). Statistical analysis was performed using unpaired two-tailed Student`s t-test (b, c) with *P < 0.05: **P < 0.01; ***P < 0.001. Source data and exact P values are provided in the Source data file.

Ring et al., Supplementary Figure 9

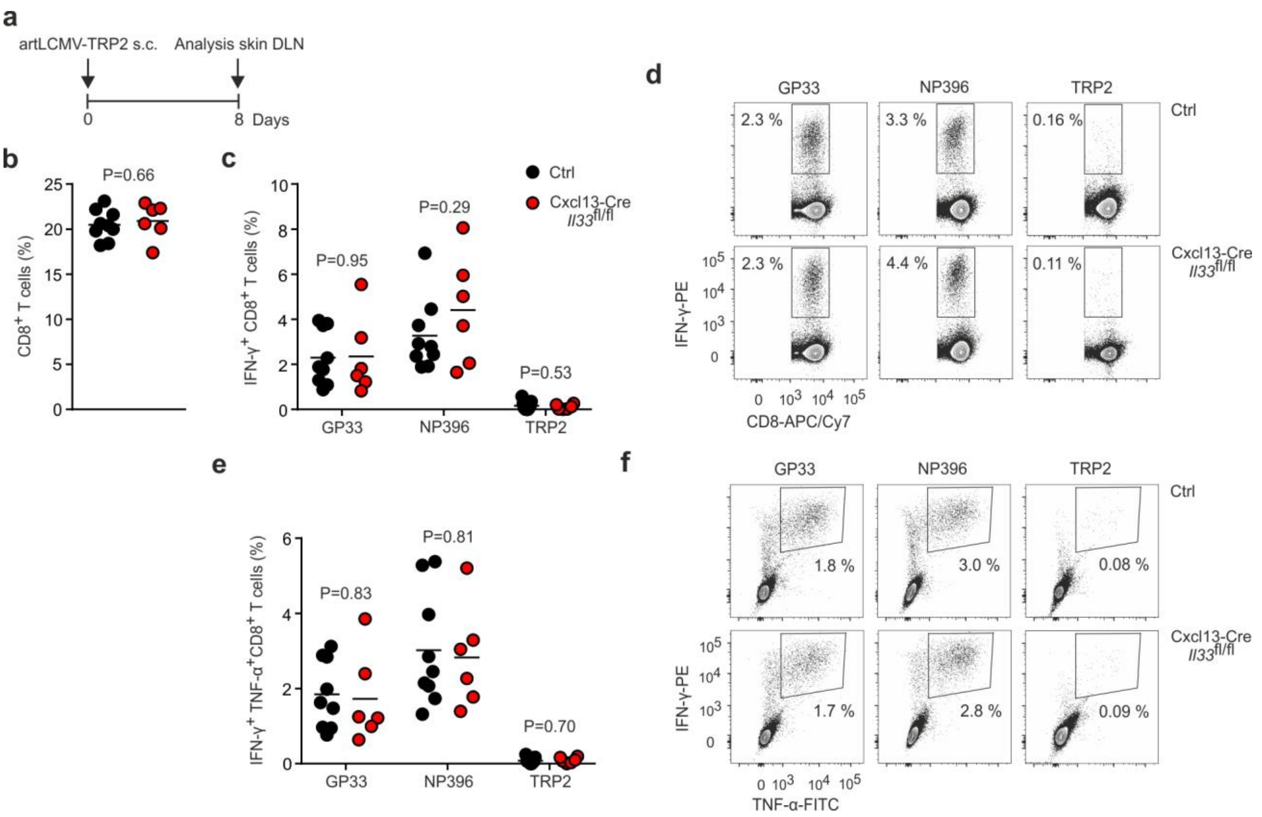

Supplementary Figure 9. FSC-specific IL-33 ablation does not impede CD8$^+$ T cell responses after s.c. artLCMV-TRP2 immunization. a, Cxc13-Cre *Il33*$^{fl/fl}$ or Cre-negative littermate control (Ctrl) mice were immunized s.c. with artLCMV-TRP2. CD8$^+$ T cell responses were analyzed in the skin draining lymph node (SDLN) on day 8 post immunization. **b,** Frequency of CD8$^+$ T cell in the SDLN. **c-f,** Flow cytometric analysis of IFN-γ$^+$ (c) and IFN-γ$^+$ TNF-α$^+$ (e) CD8$^+$ T cells after *ex vivo* stimulation with the LCMV-specific peptides GP33 and NP396 or the TRP2 peptide. Representative plots are shown for IFN-γ$^+$ (d) and IFN-γ$^+$ TNF-α$^+$ (f) CD8$^+$ T cells. Dots represent individual mice and lines indicate mean values. Pooled data from two independent experiments with n=6 (Ctrl) and n=9 (Cxc13-Cre *Il33*$^{fl/fl}$) mice. Statistical analysis was performed using unpaired two-tailed Student`s t-test (b, c, e) with *P < 0.05: **P < 0.01; ***P < 0.001. Source data are provided in the Source data file.

Ring et al., Supplementary Table 1. Antibodies for flow cytometry.

Antibody	Clone	Conjugate	Source, Cat no	Dilution
**Mouse:**				
CD106 (VCAM-1)	429	BV711	BD Biosciences	1:100
CD11b	M1/70	APC/Cy7	BioLegend	1:200
CD11c	N418	APC/Cy7, PE/Cy7	BioLegend	1:200
CD19	eBio1D3	PE/Cy7	eBioscience	1:100
CD279 (PD-1)	J43	BV421	BioLegend	1:100
CD26	H194-112	PE/Cy7	BioLegend	1:200
CD31	390	PerCP/Cy5, PE/Cy7	BioLegend	1:100
CD34	RAM34	BV421	BioLegend	1:100
CD4	RM4-5	BV605	BioLegend	1:100
CD45	30-F11	PerCP, PE/Cy7	BioLegend	1:100
CD45.1	A20	PE/Cy7	BioLegend	1:100
CD45.2	104	BV510, BV605	BioLegend	1:100
CD54 (ICAM-1)	3E2	BV421	BD Biosciences	1:100
CD62L	MEL-14	PerCP/Cy5.5	BioLegend	1:100
CD69	H1.2F3	FITC	BioLegend	1:100
CD8a	53-6.7	APC, APC/Cy7, BV786, BV711	BioLegend	1:100
Eomes	DAN11MAG	AF488	eBioscience	1:100
F4/80	BM8	AF647	BioLegend	1:100
FoxP3	FJK-165	PE	eBioscience	1:100
Granzyme B	GB11	AF647	BioLegend	1:100
H-2D^{b}/ H-2K^{b} (MHC I)	28-8-6	PE/Cy7	BioLegend	1:100
I-A/ I-E	M5/114.15.2	AF700	BioLegend	1:200
IFN-γ	XMG1.2	PE	BioLegend	1:50
KLRG1	2F1	BV421	BioLegend	1:100
LCMV-NP	VL-4	A546/ AF647	BioXCell, in house conjugation	1:100
Ly6C	HK1.4	PerCP	BioLegend	1:100
Ly6G	1A8	PE	BioLegend	1:400
PDPN	8.1.1	APC, APC/Cy7	BioLegend	1:100
Sca-1 (Ly6A/E)	D7	BV605	BioLegend	1:400
ST2 (Il1r1l)	DJ8	FITC	MD Bioproducts	1:100
T-bet	4B10	PE/Cy7	BioLegend	1:100
Ter119	Ter-119	BV510, PE/Cy7	BioLegend	1:100
TNF-α	MP6-XT22	FITC	BioLegend	1:50
TOX	TXRX10	eFluor660	eBioscience	1:100
Vα2	B20.1	APC	BD Biosciences	1:100
Vβ8.1/ Vβ8.2	MR5-2	PE	BioLegend	1:100
**Human:**				
CD90 (Thy1)	5E10	PerCP	BioLegend	1:100
PDPN	NZ-1.3	PE	eBioscience	1:100
CD19	HIB19	PE/Cy7	BioLegend	1:100
CD3	OKT3	BV605	BioLegend	1:100
HLA-DR	L243	PerCP/Cy5.5	BioLegend	1:100
CD14	HCD14	FITC	BioLegend	1:100
CD16	3G8	APC/Cy7	BioLegend	1:100
CD11c	Bu15	APC	BioLegend	1:100
CD123	9F5	BV711	BD Biosciences	1:200
