# Supplementary material for: Viral vector-mediated reprogramming of the fibroblastic tumor stroma sustains curative melanoma treatment
Source: Nat Commun. 2021 Aug 5;12:4734. doi: 10.1038/s41467-021-25057-w (PMC8342618; doi:10.1038/s41467-021-25057-w)
Supplement: Supplementary file 2 — Reporting Summary [file 41467_2021_25057_MOESM2_ESM.pdf]

nature research

Corresponding author(s): Burkhard Ludewig, Lukas Flatz

Last updated by author(s): Jul 5, 2021

Reporting Summary

Nature Research wishes to improve the reproducibility of the work that we publish. This form provides structure for consistency and transparency in reporting. For further information on Nature Research policies, see our Editorial Policies and the Editorial Policy Checklist.

Statistics

For all statistical analyses, confirm that the following items are present in the figure legend, table legend, main text, or Methods section.

n/a	Confirmed	
☐	☒	The exact sample size (n) for each experimental group/condition, given as a discrete number and unit of measurement
☐	☒	A statement on whether measurements were taken from distinct samples or whether the same sample was measured repeatedly
☐	☒	The statistical test(s) used AND whether they are one- or two-sided<br>*Only common tests should be described solely by name; describe more complex techniques in the Methods section.*
☒	☐	A description of all covariates tested
☐	☒	A description of any assumptions or corrections, such as tests of normality and adjustment for multiple comparisons
☐	☒	A full description of the statistical parameters including central tendency (e.g. means) or other basic estimates (e.g. regression coefficient) AND variation (e.g. standard deviation) or associated estimates of uncertainty (e.g. confidence intervals)
☐	☒	For null hypothesis testing, the test statistic (e.g. F, t, r) with confidence intervals, effect sizes, degrees of freedom and P value noted<br>*Give P values as exact values whenever suitable.*
☒	☐	For Bayesian analysis, information on the choice of priors and Markov chain Monte Carlo settings
☒	☐	For hierarchical and complex designs, identification of the appropriate level for tests and full reporting of outcomes
☒	☐	Estimates of effect sizes (e.g. Cohen's d, Pearson's r), indicating how they were calculated

Our web collection on statistics for biologists contains articles on many of the points above.

Software and code

Policy information about availability of computer code

Data collection	FACSDiva (BD Bioscience, v8.0.1), FACSChorus (BD Biosciences, v1.3), ZEN (Zeiss, v14.0.18.201), Imaris v9, The QuantStudioTM Design & Analysis Software v1.5.1, NovaSeq 6000 sequencing system (Illumina)
Data analysis	FlowJo (Treestar Inc., v10), Prism (Graphpad, v8.4.3), R Studio (v 3.6.1), Cell Ranger (v 3.0.2), R packages running in R v3.6.1: scater (v1.14.1), Seurat (v3.1.1), clusterProfiler (v.3.16.0), SeuratWrappers (v.0.1.0), runDiffusionMap function (v1.16.2), Imaris version 9 (Bitplane)

For manuscripts utilizing custom algorithms or software that are central to the research but not yet described in published literature, software must be made available to editors and reviewers. We strongly encourage code deposition in a community repository (e.g. GitHub). See the Nature Research guidelines for submitting code & software for further information.

Data

Policy information about availability of data

All manuscripts must include a data availability statement. This statement should provide the following information, where applicable:

- Accession codes, unique identifiers, or web links for publicly available datasets
- A list of figures that have associated raw data
- A description of any restrictions on data availability

ScRNA-seq data are available in ArrayExpress database (accession numbers E-MTAB-9407). Ensembl GRCm38.94 was used as reference to build index files for alignments in scRNA-seq analysis. Further information and requests for resources should be directed to and will be fulfilled by the lead contacts.

Field-specific reporting

Please select the one below that is the best fit for your research. If you are not sure, read the appropriate sections before making your selection.

- [x] Life sciences
- [ ] Behavioural & social sciences
- [ ] Ecological, evolutionary & environmental sciences

For a reference copy of the document with all sections, see nature.com/documents/nr-reporting-summary-flat.pdf

Life sciences study design

All studies must disclose on these points even when the disclosure is negative.

Sample size	No sample-size calculation was performed; sample size were determined to be adequate based on the reproducibility between independent experiments.
Data exclusions	No data points were excluded
Replication	Experiments were repeated at least two times. Animal experiments were repeated with sufficient animals per group to demonstrate statistical significance. All experiments were reliably reproduced.
Randomization	Subjects were randomly assigned to experimental groups. For animal experiments, six to ten weeks old female mice were age matched and randomly assigned to experimental groups.
Blinding	Blinding was not performed in this study. Data analysis was strictly quantitative, with objective outcomes therefore blinding was not relevant for this study.

Reporting for specific materials, systems and methods

We require information from authors about some types of materials, experimental systems and methods used in many studies. Here, indicate whether each material, system or method listed is relevant to your study. If you are not sure if a list item applies to your research, read the appropriate section before selecting a response.

Materials & experimental systems

n/a	Involved in the study	
[ ]	[x]	Antibodies
[ ]	[x]	Eukaryotic cell lines
[x]	[ ]	Palaeontology and archaeology
[ ]	[x]	Animals and other organisms
[ ]	[x]	Human research participants
[x]	[ ]	Clinical data
[x]	[ ]	Dual use research of concern

Methods

n/a	Involved in the study	
[x]	[ ]	ChIP-seq
[ ]	[x]	Flow cytometry
[x]	[ ]	MRI-based neuroimaging

Antibodies

Antibodies used

The following antibodies were used (name, clone, supplier, catalog number, dilution):
Mouse:
anti-CD106 (VCAM-1)-BV711 429 BD Biosciences 740675, 1:100
anti-CD11b-APC/Cy7 M1/70 BioLegend 101225, 1:200
anti-CD11c-APC/Cy7, PE/Cy7 N418 BioLegend 117324, 117318, 1:200
anti-CD19-PE/Cy7 eBio1D3 eBioScience 25-0193-82, 1:100
anti-CD279 (PD-1)-BV421 J43 BioLegend 562584, 1:100
anti-CD26-PE/Cy7 H194-112 BioLegend 137810, 1:200
anti-CD31-PerCP/Cy5, PE/Cy7 390 BioLegend 102420, 102418, 1:100
anti-CD34-BV421 RAM34 BioLegend 119321, 1:100
anti-CD4-BV605 RM4-5 BioLegend 100548, 1:100
anti-CD45-PE/Cy7 30-F11 BioLegend 103114, 1:100
anti-CD45.1-PE/Cy7 A20 BioLegend 110730, 1:100
anti-CD45.2-BV510, BV605 104 BioLegend 109838, 109841, 1:100
anti-CD54 (ICAM-1)-BV421 3E2 BD Biosciences 553252, 1:100
anti-CD62L-PerCP/ Cy5.5 MEL-14 BioLegend 104432, 1:100
anti-CD69-FITC H1.2F3 BioLegend 104506, 1:100
anti-CD8a-APC, APC/Cy7, BV711 53-6.7 BioLegend 100712, 100714, 100748, 1:100
anti-Eomes-A488 DAN11MAG eBioscience 53-4875-82, 1:100

anti-F4/80-AF647 BM8 BioLegend 123114, 1:100
anti-FoxP3-PE FJK-165 eBioScience 12577382, 1:100
anti-Granzyme B-AF647 GB11 BioLegend 515406, 1:100
anti-H-2Db/ H-2Kb (MHC I)-PE/Cy7 28-8-6 BioLegend 114616, 1:100
anti-I-A/ I-E-AF700 M5/114.15.2 BioLegend 107621, 1:200
anti-IFN-γ-PE XMG1.2 BioLegend 505808, 1:50
anti-KLRG1-BV421 2F1 BioLegend 138414, 1:100
anti-LCMV-NP-A546/ AF647 VL-4 BioXCell BE0106, in house conjugation 53044, A20186, 1:100
anti-Ly6C-PerCP HK1.4 BioLegend 128028, 1:100
anti-Ly6G-PE 1A8 BioLegend 127608, 1:400
anti-PDPN-APC, APC/Cy7 8.1.1 BioLegend 127410, 127418, 1:100
anti-Sca-1 (Ly6A/E)-BV605 D7 BioLegend 108134, 1:400
anti-ST2 (Il1r1l)-FITC DJ8 MD Bioproducts 101001F, 1:100
anti-T-bet-PE/Cy7 4B10 BioLegend 644810, 1:100
anti-Ter119-BV510, PE/Cy7 Ter-119 BioLegend 116237, 116222, 1:100
anti-TNF-α-FITC MP6-XT22 BioLegend 506304, 1:50
anti-TOX-eFluor660 TXRX10 eBioscience 50-6502-82, 1:100
anti-Vα2-APC B20.1 BDBiosciences 127810, 1:100
anti-Vβ8.1/ Vβ8.2-PE MR5-2 BioLegend 140104, 1:100

Tetramer:
PE-labelled H-2Kb-TRP2 180-188 tetramer MBL, Woburn, MA TB-5004-1, 1:100

Human:
anti-CD90 (Thy1)-PerCP 5E10 BioLegend 328117, 1:100
anti-PDPN-PE NZ-1.3 eBioscience 12-9381-42, 1:100
CD19-PE/Cy7 HIB19 BD Biosciences 557835, 1:100
CD3-BV605 OKT3 BioLegend 317322, 1:100
HLA-DR-PerCP/Cy5.5 L243 BioLegend 307629, 1:100
CD14-FITC HCD14 BioLegend 345784, 1:100
CD16-APC/Cy7 3G8 BioLegend 302018, 1:100
CD11c -APC Bu15 BioLegend 301613, 1:100
CD123-BV711 9F5 BD Biosciences 563161, 1:200

Immunohistochemistry:
anti-PDPN, eBio8.1.1 BioLegend 127402, 1:200
anti-PDPN-bio eBio8.1.1 BioLegend 13-5381-82, 1:200
anti-GFP Aves GFP-1020, 1:1000
anti-mIL-33 R&D systems AF3626, 1:500
anti-LCMV-NP-AF647 VL-4 BioXCell BE0106, in house conjugation A20186, 1:200
anti-αSMA-Cy3 1A4 Sigma C6198, 1:400
CD90-FITC 5E10 BioLegend 328108, 1:100
goat anti-chicken-IgG-AF488 Thermofisher #A-11039, 1:1000
goat anti-syrian hamster-IgG-Dylight549 Jackson Immunotools 107-505-142, 1:1000
donkey anti-goat IgG-AF647 Jackson Immunotools 705-607-003, 1:500
Streptavidin-AF488 Jackson Immunotools 016-540-084, 1:1000

Validation: All mouse and human antibodies came from commercial vendors and have been validated by the manufacturers on their official websites.

Eukaryotic cell lines

Policy information about cell lines

Cell line source(s): The tumor cell line B16F10 cells was purchased from ATCC. BHK-21 (generated by L. Flatz and D. Pinschewer) and HEK293 (Hookipa Pharma) cells are stable transfectants carrying plasmid M369 that expresses the LCMV-glycoprotein (LCMV-GP) cDNA.

Authentication: Cell lines were not authenticated in out laboratory.

Mycoplasma contamination: All cell lines were tested negative for mycoplasma.

Commonly misidentified lines (See ICLAC register): No misidentified cell lines were used.

Animals and other organisms

Policy information about studies involving animals; ARRIVE guidelines recommended for reporting animal research

Laboratory animals: BAC-transgenic C57BL/6N-Tg(Cxcl13-Cre/Tdtomato)719Biat (Cxcl13-Cre/Tdtomato) mice were generated using BAC RP23-57A17

(Invitrogen), transgene integration was achieved using Red/ET recombination technologies (Gene bridges). C57BL/6N (B6), C57BL/6N, R26R–enhanced yellow fluorescent protein (EYFP; B6.129X1-Gt[ROSA]26Sortm1[EYFP]Cos/J) mice were purchased from Charles River (Sulzfeld, Germany). The Il33 targeting vector to generate the Il33fl/fl mice was generated by the trans-NIH Knock-Out Mouse Project (KOMP Project ID CSD88909) and obtained from the KOMP Repository (www.komp.org). To ablate Il33 expression in a distinct FSC subset, Cxcl13-Cre mice were crossed with Il33fl/fl mice. Cxcl13-Cre mice were bred to R26R-EYFP mice to generate heterozygous Cxcl13-Cre R26R-EYFP. Transgenic mice expressing the P14 T cell receptor specific for the H-2Db-restricted epitope GP33-41 and CD45.1 as congenic marker were used as organ donors for adoptive CD8+ T cell transfer experiments. P14 TCR transgenic mice crossed to Il1rl1 (ST2)-deficient mice. ST2-/- P14 mice were used under MTA from A. McKenzie (MRC, Cambridge, UK). All mice were housed in the Institute of Immunobiology, Kantonsspital St. Gallen under specific-pathogen-free (SPF) conditions at 22 °C and 30–70% humidity in a 12/12-h light/dark cycle and provided ad libitum access to food and water. Experiments were performed with 6 to 10 weeks old female mice.

Wild animals	None
Field-collected samples	None
Ethics oversight	All mouse experiments were performed under a study protocol approved by Swiss federal and St. Gallen cantonal ethical committees under permission numbers SG08/17, SG01/18, SG07/19, SG04/20 and SG01/20.

Note that full information on the approval of the study protocol must also be provided in the manuscript.

Human research participants

Policy information about studies involving human research participants

Population characteristics	Skin specimens were taken from healthy patients undergoing abdominoplasty (female and male, age 18 to 61). Melanoma tissue was obtained from punch biopsies from a primary tumor of a melanoma patient (female, age 58). Patient-matched PBMCs or PBMCs from healthy volunteers were obtained. All patients have signed informed consent.
Recruitment	Patient samples were obtained from patients of the Department of Plastic Reconstructive Surgery, the Department of Dermatology and the Institute of Pathology at the Kantonsspital St. Gallen and healthy volunteers. There was no self-selection bias or investigator selection bias in determining which subjects were included in the study or in the data analysis.
Ethics oversight	The study has been approved by the ethics committee of Eastern Switzerland (EKOS16-079, BASEC Nr. 2016-00998) and all patients provided a written informed consent.

Note that full information on the approval of the study protocol must also be provided in the manuscript.

Flow Cytometry

Plots

Confirm that:

- [x] The axis labels state the marker and fluorochrome used (e.g. CD4-FITC).
- [x] The axis scales are clearly visible. Include numbers along axes only for bottom left plot of group (a 'group' is an analysis of identical markers).
- [x] All plots are contour plots with outliers or pseudocolor plots.
- [x] A numerical value for number of cells or percentage (with statistics) is provided.

Methodology

Sample preparation	A description of sample preparation for the flow cytometry is detailed in the methods section.
Instrument	LSR Fortessa 2 BD Bioscience, FACS Melody BD Bioscience
Software	FACS Diva was used to collect the data and FlowJo (Treestar Inc.) to analyze the data. For cell sorting for scRNA seq, FACSChorus was used to set up cell sorting. R studio was used to analyze transcriptomic data.
Cell population abundance	Cells were checked for purity of sorted populations after flow cytometry assisted cell sorting using LSR Fortessa 2.
Gating strategy	For all flow cytometric analysis, cells were first gated on FSC/SSC to exclude cell debris following by FSC-A/FSC-H to exclude doublets. Dead cells were excluded from analysis by gating on viability dye negative staining. Gating strategy for identifying cell population analyzed in this study is exemplifying in the Supplementary Figures.

- [x] Tick this box to confirm that a figure exemplifying the gating strategy is provided in the Supplementary Information.
